# Biomechanical comparison of three minimally invasive fixations for unilateral pubic rami fractures

**DOI:** 10.1186/s12891-020-03604-8

**Published:** 2020-09-04

**Authors:** Yong Zhao, Yupeng Ma, Dexin Zou, Xiujiang Sun, Gong Cheng, Wei Lian, Shengjie Dong, Yuchi Zhao, Wenqing Qu, Hao Wu

**Affiliations:** 1 Orthopaedics Department, Yantai Shan Hospital, 91#, Jiefang Road, Yantai, 264008 Shandong Province People’s Republic of China; 2 CT/MR Department, Yantai Shan Hospital, 91#, Jiefang Road, Yantai, 264008 Shandong Province People’s Republic of China

**Keywords:** Pelvis, Pubic fracture, Minimal invasion, Internal fixation, Biomechanics

## Abstract

**Background:**

To compare the mechanical characteristics of a percutaneous superior pubic intramedullary screw, percutaneous bridging plate and percutaneous screw-rod system of the anterior ring for the treatment of unilateral vertical pubic fractures to provide a reference for clinical application.

**Methods:**

A finite element model of pelvic anterior ring injury (unilateral vertical pubic fracture) was produced. The fractures were fixed with a percutaneous superior pubic intramedullary screw, percutaneous bridging plate and percutaneous screw-rod system of the anterior ring and their combinations in 5 types of models. The fracture stabilities under vertical, bilateral and anterior-posterior load were quantified and compared based on the displacement of the hip joints’ midpoint as quantificational index of fracture stability.

**Results:**

In the condition of bilateral and anterior-posterior load, the vertical, bilateral and anterior-posterior displacements of the hip joints’ midpoint of different models were significantly different respectively. In general, the displacements of the 5 pelvic anterior ring fixations were ranked from maximum to minimum as follows: bridging plate, pelvic anterior screw-rod system, combination of bridging plate and pelvic anterior screw-rod system, superior pubic intramedullary screw and combination of superior pubic intramedullary screw and pelvic anterior screw-rod system.

**Conclusion:**

For the fixation in unilateral pubic fractures of pelvic ring injury, the percutaneous superior pubic intramedullary screw is optimal, the percutaneous pelvic anterior screw-rod system is the second choice, and percutaneous bridging plate ranks the third. The percutaneous pelvic anterior screw-rod system can significantly increase fixation stability of the percutaneous superior pubic intramedullary screw and the percutaneous bridging plate.

## Background

In order to preserve life, it is impossible to perform time-consuming final reduction and internal fixation with more surgical trauma and much blood loss, at the early stage of treatment of pelvic fracture with combined injuries of important organs, blood vessels, and nerves. Furthermore, due to the distinctive anatomical and physiological characteristics of the pelvis, the pelvic injury has become timeworn when the patient’s general conditions turn to be stable. In this case, the difficulty of reduction and fixation has increased significantly. The deformity is difficult to be corrected, and the risk of intraoperative vascular and neurologic injury significantly increases. This situation is common in Tile C pelvic fracture with poor stability caused by severe injury. Therefore, in order to minimize the initial mortality rate and late disability rate of pelvic injury, it is particularly essential to perform early, rapid and minimally invasive replacement and internal fixation after an injury and on the precondition that the body can tolerate it.

The importance of reconstruction of posterior ring stability is self-evident in the treatment of pelvic fractures. However, in terms of some Tile B and all Tile C pelvic injuries, it has been proved that only posterior ring fixation cannot satisfy the requirements of maintaining bone position. Previous biomechanical researches have demonstrated that the stability of any posterior ring-internal fixator complex does not match the normal intact pelvis [1, 2]. Therefore, the combined fixation of anterior and posterior rings is undoubtedly a necessary means to enhance the effect of internal fixation. In other words, the fixation of the anterior pelvic ring plays a vital role in the reconstruction of the whole pelvic mechanical characteristics.

So far, primary minimally invasive treatments of the final internal fixation for the pelvic anterior ring includes percutaneous superior pubic intramedullary screw, percutaneous bridging plate and percutaneous screw-rod system of the anterior ring [3]. How to optimize the fixation effect of abovementioned minimally invasive anterior ring internal fixation has positive significance for the treatment of unstable pelvic fractures. Based on this, the authors carried out a biomechanical comparative study among the aforementioned three kinds of anterior ring minimally invasive internal fixations, in order to formulate a rapid, accurate and minimally invasive clinical treatment project.

## Methods

The ethics committee of Yantai Shan Hospital approved the study. Informed consents were obtained from the individual participant included in the study.

In this study, the three-dimensional finite element model of pelvis established by our series of researches was used, and the related parameters were in line with the relevant literature [1, 2, 4]. The central sagittal plane of the right superior and inferior ramus of the pubis in the normal pelvic model was defined as the fracture surface, and the type unilateral longitudinal pubic rami fracture was simulated.

According to the size of screw, plate and screw rod, parametric design models was established to simulate the operation modes of the percutaneous bridging plate, anterior ring screw-rod system and superior pubic intramedullary screw to fix the pubic superioris. The superior pubic intramedullary screw was a cannulated screw with a diameter of 7.3 mm and a length of 100 mm. The screw was placed retrograde and located in the center of the safe area of the superior ramus of pubis, and the screw thread passed through the fracture line.

The percutaneous bridging plate was a reconstructed plate on the surface of the pelvic anterior ring. Its proximal part was located nearby right anterior superior iliac spine, and the distal portion was located at superior ramus of pubis beside the pubic symphysis. Three cortical screws with 3.5 mm diameter and 15-32 mm length were placed at the plate proximal and distal ends respectively.

The screw of the anterior ring screw-rod system was 6.5 mm in diameter and 80 mm in length. It was inserted from the anterior inferior iliac spine and pointed to the posterior inferior iliac spine. Boolean operations were performed on the pelvic bone and internal fixators to form fixation models.

**Fig. 1:**
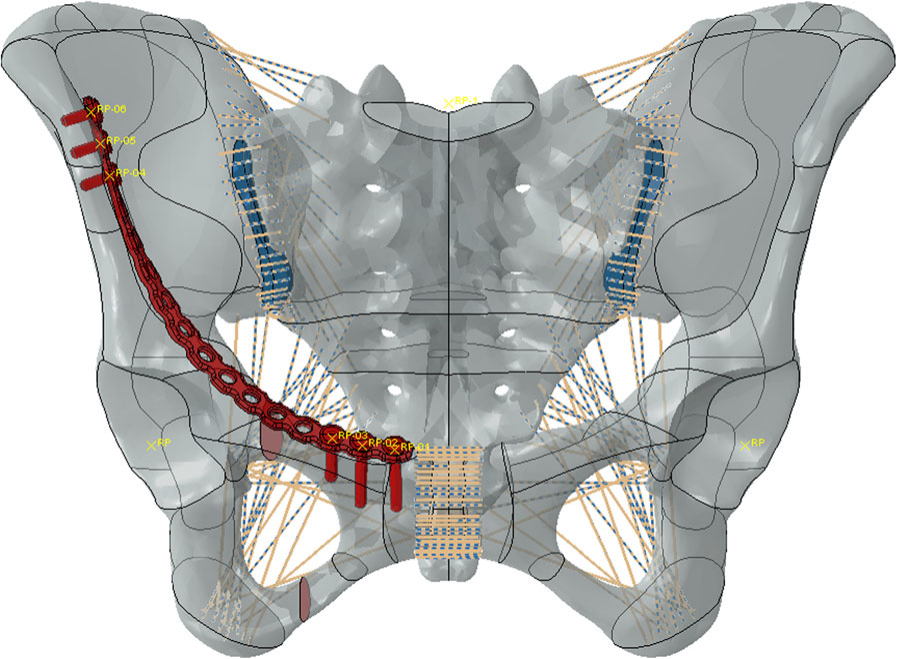
Right percutaneous anterior ring bridging plate (P)

**Fig. 2:**
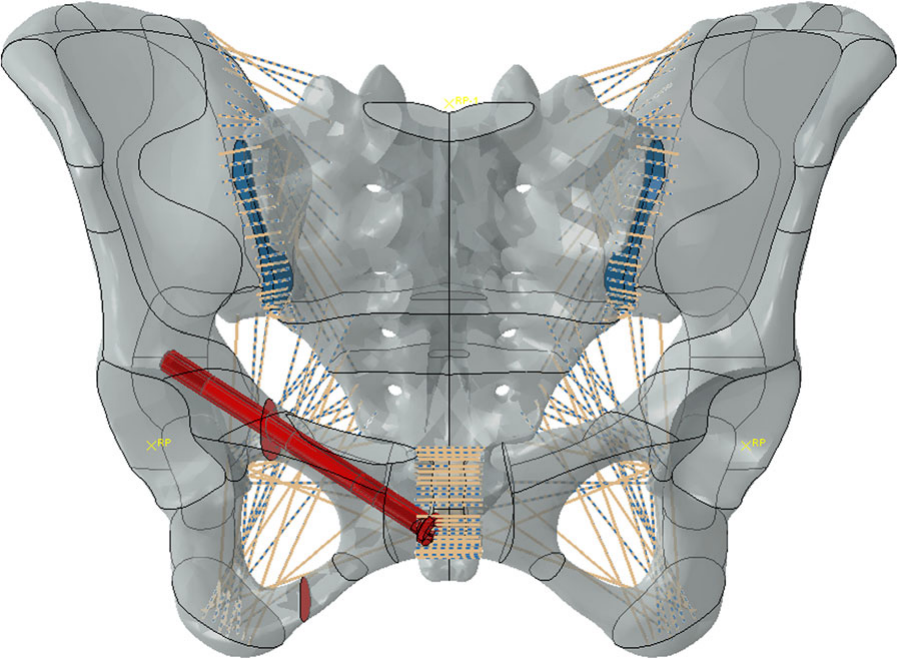
Right percutaneous superior pubic intramedullary screw (S)

**Fig. 3:**
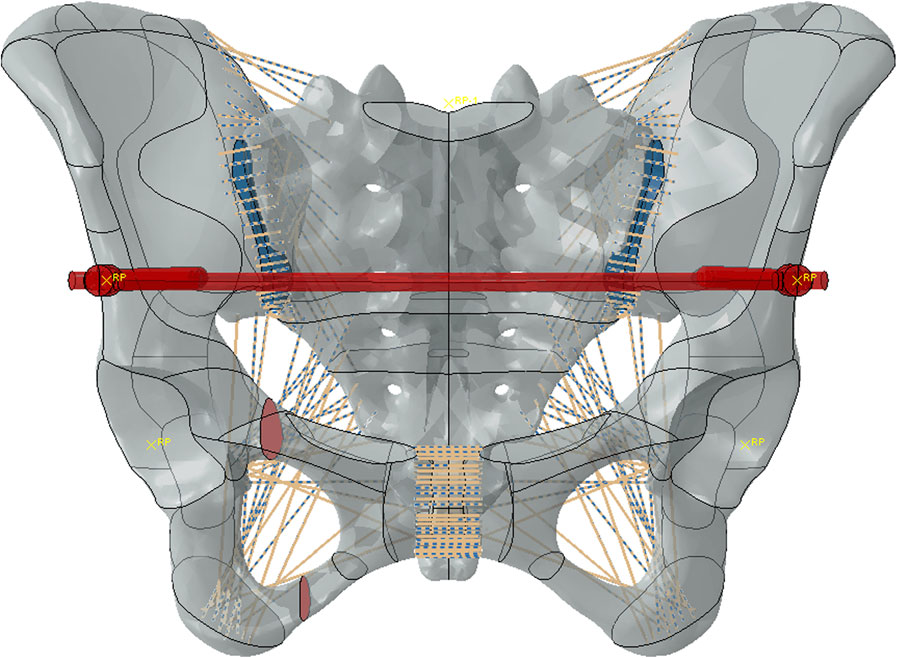
Percutaneous anterior ring screw-rod system (R)

**Fig. 4:**
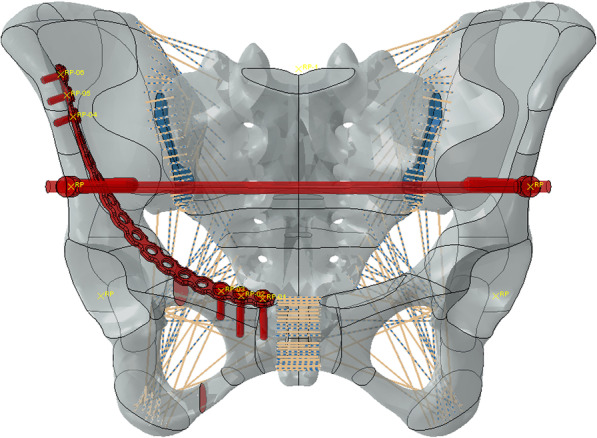
Combination of right percutaneous anterior ring bridging plate and percutaneous anterior ring screw-rod system (P + R)

**Fig. 5:**
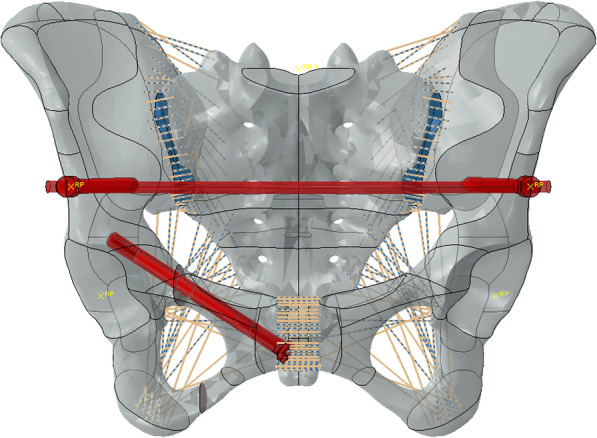
Combination of right percutaneous superior pubic intramedullary screw and percutaneous anterior ring screw-rod system (S + R)

Simulated fixation modes were as follows: right percutaneous anterior ring bridging plate (P), right percutaneous superior pubic intramedullary screw (S), percutaneous anterior ring screw-rod system (R), the combination of right percutaneous anterior ring bridging plate and percutaneous anterior ring screw-rod system (P + R), the combination of right percutaneous superior pubic intramedullary screw and percutaneous anterior ring screw-rod system (S + R). (Table 1) (Figs. 1, 2, 3, 4 and 5).

**Table 1:** The minimally invasive internal fixation models for unilateral pubic fractures

Fixation models (abbreviation)	Five fixation modes (specific description)
P	Right percutaneous anterior ring bridging plate
S	Right percutaneous superior pubic intramedullary screw
R	Percutaneous anterior ring screw-rod system
P + R	Combination of right percutaneous anterior ring bridging plate and percutaneous anterior ring screw-rod system
S + R	Combination of right percutaneous superior pubic intramedullary screw and percutaneous anterior ring screw-rod system

Tie constraints were applied between the bone-implant interfaces except for the cannulated screw stem regions where the frictionless sliding contact was applied. Penalty contact with a friction coefficient of 0.3 was applied between the interaction surfaces of fractures. The longitudinal downward load, anteroposterior load and transverse load were simulated to imposed to pelvis respectively, and all of them were 600 N.

Under three different loads, the displacements of the virtual right hip midpoint in vertical, transverse and anteroposterior directions were taken as the evaluation index of stability. The smaller the displacement, the better the stability.

## Results

In the simulated bipedal standing situation, there was no significant difference among the vertical, transverse and anteroposterior displacements of the virtual hip midpoint in the models of bridging plate, superior pubic intramedullary screw, anterior ring screw-rod system and their combined fixations. Similarly, the results of the simulation of standing on 1 foot were almost the same as those of the simulation of standing on 2 feet mentioned above.

Under the simulated anteroposterior loading condition, there were obvious differences among the virtual hip midpoint’s displacements in anteroposterior, transverse and vertical directions in three models of pelvic anterior ring single fixation, in which bridging plate was the largest, anterior ring screw-rod system was the second, and the intramedullary screw was the smallest. Comparing the models of single with combined fixations, it demonstrated that the assistant of the anterior ring screw-rod system significantly reduced the displacement of three directions in single bridging plate fixation and sole superior pubic intramedullary screw fixation, and the displacement of the model of superior pubic intramedullary screw combined with anterior ring screw-rod system was the smallest.

Under the simulated left-right loading condition, there were obvious differences among the virtual hip midpoint’s displacements in anteroposterior, transverse and vertical directions in three models of pelvic anterior ring single fixation, in which bridging plate was the largest, and anterior ring screw-rod system was the second, and intramedullary screw was the smallest. Comparing the models of single and combined fixations, it illustrated that the assistant of the anterior ring screw-rod system significantly reduced the displacements of three directions in single bridging plate fixation and the displacements of left-right and anteroposterior directions in single intramedullary screw fixation, however there is no effect on the displacement of vertical direction in single intramedullary screw fixation. The displacement in the model of the combination of superior pubic intramedullary screw and anterior ring screw-rod system was the smallest.

Generally speaking, under simulated anteroposterior and transverse loading conditions, the multidirectional displacements in all models were ranked from small to large as follows: the combination of superior pubic intramedullary screw and anterior ring screw-rod system, superior pubic intramedullary screw, the combination of bridging plate and anterior ring screw-rod system, anterior ring screw-rod system and pelvic anterior bridging plate. (Tables 2, 3 and 4).

**Table 2:** The multidirectional displacement under vertical load (mm)

	Transverse displacement	Vertical displacement	Anteroposterior displacement
P	0.06	1.14	1.03
S	0.02	1.14	1.06
R	0.01	1.11	1.04
P + R	0.01	1.11	1.04
S + R	0.01	1.11	1.04

**Table 3:** The multidirectional displacement under anteroposterior load (mm)

	Transverse displacement	Vertical displacement	Anteroposterior displacement
P	3.69	0.98	3.57
S	2.50	0.70	2.74
R	2.79	0.88	3.04
P + R	2.66	0.79	2.90
S + R	2.28	0.63	2.52

**Table 4:** The multidirectional displacement under left-right load (mm)

	Transverse displacement	Vertical displacement	Anteroposterior displacement
P	3.49	0.33	2.31
S	0.69	0.04	0.44
R	1.49	0.26	0.96
P + R	1.38	0.14	0.84
S + R	0.58	0.04	0.37

## Discussion

Antegrade and retrograde percutaneous pubic intramedullary screw technique avoids extensive exposure during operation via ilioinguinal approach etc., and achieves ideal clinical results. However, because of the risk of bladder injury, iliac artery and vein injury, and hip joint involvement, the cannulated screw is a relatively safe choice [5–7]. If the pelvic anterior ring is anatomically variable, the intramedullary screws should be placed more carefully. Although longer screw can provide better stability, the risk of cannulated screw breakage is significantly increased when the screw is too long (length > 100 mm) and too thin (diameter < 6.5 mm). Therefore, the diameter of the superior pubic branch less than 6.5 mm is also a contraindication of intramedullary cannulated screw technique [8]. As the pubis is close to the bladder, iliac artery and iliac vein, retrograde screw placement based on the easily accessible pubic tubercle are safer. Weatherby et al . [9] believe that retrograde screws were having a lower failure rate for fractures medial to the lateral border of the obturator foramen.

Compared with conventional plate internal fixation, anterior ring bridging plate has the advantage of minimally invasive percutaneous implantation, which effectively avoids the pain of surgical site and incision complications, [10] and significantly reduces the incidence of iatrogenic injury such as vascular and nervous system. Because of the low requirement for the anatomical shape of the anterior pelvic ring, the technique has broad indications. It can be used as an effective supplement to the superior pubic intramedullary screw. However, due to the long plate and few screw, its biomechanical performances still need to be further studied.

As an ‘inbuilt external fixator’, the pelvic anterior screw-rod system creates the principle of fixation above the acetabulum in order to stabilize the anterior pelvic ring by minimal screw and more minimally invasive means and to help fracture reduction by lateral compression and stretching. Although a biomechanical study concluded that the anterior subcutaneous pelvic ring fixator had no biomechanical advantage over the traditional external pelvic fixation, [11] the fixation located in the body does not affect sitting, standing and walking, and is suitable for obese patients especially for severe patients with abdominal organ injury [10]. However, the screw placement requires higher technique, and there is a risk of iatrogenic injury of anterolateral femoral cutaneous nerve and hip joint capsule, with a higher incidence of heterotopic ossification [12]. The internal fixation needs to be removed by a second operation. In addition, bilateral fixation is necessary for both unilateral and bilateral pubic fractures, so the biomechanical characteristics of this kind of fixation and its possible impacts on pubic symphysis deserve in-depth study.

Thus, three kinds of pelvic anterior ring minimally invasive internal fixation methods have their own pros and cons. Further studies are needed about how to choose an effective and safe fixation method according to the specific traumatic condition, available hardware and technical level, and maximize the biomechanical advantages of various anterior ring internal fixations, so as to achieve the best effect of minimally invasive internal fixation, which helps to define the respective indications of the anterior ring minimally invasive internal fixation methods, pre-plan the operation pertinently and effectively shorten the preoperative preparation and operation time.

In this study, it was found that under the simulated bipedal standing state, there were no significant differences in the vertical, bilateral and anteroposterior stabilities of the fractures between the models of the percutaneous superior pubic intramedullary screw, percutaneous bridging plate and percutaneous anterior ring screw-rod system and their combined fixations. Then, the state of standing on a single foot was simulated, and the results were similar to those of the state of standing on two feet. This suggested that the anterior pelvic ring contributed little to the overall vertical stability of the pelvis. Therefore, backward loads and right-and-left loads were applied to the pelvis to simulate the mechanism of open-book and lateral injuries. By comparing the displacements of the virtual acetabular midpoint in vertical, transverse and anteroposterior directions, the translation and rotation stabilities of three kinds of anterior ring fixations and their combined fixations on coronal, sagittal and horizontal planes were determined. The results show a significant consistency. Comparing the multidirectional stabilities of three kinds of anterior ring internal fixators’ independent fixation, the percutaneous superior pubic intramedullary screw is the best, the percutaneous anterior ring screw-rod system is the second, and the percutaneous bridging plate is the worst, which shows the significant mechanical advantage of intramedullary centraxonial fixation. Although the fixation effect of anterior ring screw rod system is affected by indirect fixation across pubic symphysis, it still shows better mechanical characteristics than bridging plate with eccentric fixation. Considering the material consistency of the three internal fixators, it can not be excluded that this result may be related to the bridging plate’s ‘congenital deficiency’ of thin and flat plate and short screws.

In order to maximize the effect of internal fixation, it was planned to combine three kinds of anterior ring minimally invasive internal fixation devices in pairs. However, the superior pubic intramedullary screw and bridging plate could not be fixed simultaneously because of the limitation of anterior ring anatomy, so only the anterior ring screw-rod system was combined with the intramedullary screw and bridging plate respectively. The experiment shows that, compared with the independent fixation modes, the combined anterior ring internal fixation modes show obvious mechanical advantages, among which the combination of the superior pubic intramedullary screw and anterior ring screw-rod system achieves the most stable fixation effect. Although the combination of bridging plate and anterior ring screw-rod system is superior to their independent fixation, it is still inferior to the single superior pubic intramedullary screw. This suggests that in the clinical practice of fixing unilateral pubic fracture, superior pubic intramedullary fixation should be preferred as far as possible. In order to minimize the failure risk of internal fixation, it is suggested to combine superior pubic intramedullary fixation with anterior ring screw-rod system fixation.

However, it is undeniable that in some cases, smaller pubic diameter, a variation of pubic curvature or non anatomical reduction of pubic fractures result in the unavailability of 7.3 mm and 6.5 mm screws, so 4.5 mm or 3.5 mm diameter screws are also used by some orthopedic surgeons. As mentioned above, it was reported that the risk of cannulated screw breakage increased when the screw’s diameter was less than 6.5 m m[8]. In order to optimize the stability of fixation and reduce the risk of screw breakage effectively, we make 7.3 mm cannulated screws as the first choice for anterior column screws in clinical practice. So in this study, we did not simulate other types of screws except 7.3 mm screws. It is hoped that related research will be helpful to evaluate the effectiveness and safety of various screws of different diameters in the future.

## Conclusions

For unilateral anterior ring injury of type pelvic fracture, in general, there are obvious consistent differences in transverse, vertical and anteroposterior stabilities of the three internal fixations and their combinations; In terms of stability, the combination of percutaneous superior pubic intramedullary screw and percutaneous anterior ring screw-rod system is the best, percutaneous superior pubic intramedullary screw ranks second, percutaneous bridging plate with percutaneous anterior ring screw-rod system is the third, percutaneous anterior ring screw-rod system is the fourth, and percutaneous bridging plate is the worst; Percutaneous anterior ring screw-rod system can significantly increase the stability of percutaneous bridging plate fixation and superior pubic intramedullary screw fixation.

## Data Availability

The datasets used and/or analysed during the current study are available from the corresponding author on reasonable request.

## Abbreviations

P: In tables and figure legends are the abbreviation of right percutaneous anterior ring bridging plate.
S: In tables and figure legends are the abbreviation of the right percutaneous superior pubic intramedullary screw.
R: In tables and figure legends are the abbreviation of percutaneous anterior ring screw-rod system.
P + R: In tables and figure legends are the abbreviation of the combination of right percutaneous anterior ring bridging plate and percutaneous anterior ring screw-rod system.
S + R: In tables and figure legends are the abbreviation of the combination of right percutaneous superior pubic intramedullary screw and percutaneous anterior ring screw-rod system.
